# The effect of point-of- care ultrasonography on emergency department length of stay and CT utilization in children with suspected appendicitis

**DOI:** 10.1186/2036-7902-6-S1-A32

**Published:** 2014-01-31

**Authors:** James W Tsung, Ee T Tay, Inna Elikashvili

**Affiliations:** 1 Departments of Emergency Medicine and Pediatrics, Mount Sinai School of Medicine, New York, NY, USA

## Background

The role of clinician-performed ultrasonography for suspected appendicitis is controversial. Published data conclude that ultrasonography has high specificity to rule in the diagnosis of appendicitis with variable sensitivity to rule it out. Newer data suggest that point-of-care ultrasonography (PoCUS) may have similar test characteristics. This is the first study to examine the effect of point-of-care ultrasonography on emergency department (ED) length of stay (LOS) and CT utilization in children with suspected appendicitis.

## Objective

To evaluate the sensitivity, specificity, positive and negative likelihood ratios of PoCUS for children with suspected appendicitis and its effect on ED LOS and CT utilization.

## Methods

The study involved a prospective observational convenience sample of children with suspected appendicitis requiring imaging evaluation adhering to the STARD criteria to determine PoCUS test characteristics. Outcomes were determined by operative or pathology report in those that had appendicitis, and three week phone follow-up in those patients that were non operative. Differences in ED LOS were analyzed by ANOVA between patients who received a disposition after PoCUS, radiology US (RUS), or CT.

## Results

Among 150 enrolled patients, 50 had appendicitis (33.3%). The test performance characteristics of PoCUS, RUS, and CT are presented in Table 1. Those that had a disposition after PoCUS (N=25) had a significantly decreased mean ED LOS by one-way ANOVA (154 minutes, 95% CI 115-193) compared with those requiring Radiology US (288 minutes, 95% CI 257-319) or CT scan (487 minutes; 95% CI 434-540). Baseline CT utilization rate was 44.2% prior to study start and decreased to 27.3% during the study. There were no missed cases of appendicitis in discharged patients at 3 week phone follow-up nor negative laparotomies in those that went to the OR.

**Table 1:** Test Performance Characteristics

Imaging Modality	N	Sensitivity % (95% CI)	Specificity % (95% CI)	LR+(95% CI)	LR-(95% CI)
PoCUS	150	60 (46.2-72.4)	94 (88-97.3)	10.4 (4.63-23.35)	0.42 (0.30-0.59)

RUS	117	62.5 (47.7-75.3)	99.3 (94-99.9)	93.8. (5.9-1499)	0.38 (0.26-0.55)

CT	41	83.3 (58.4-94.7)	98.1 (84.5-99.8)	45 (2.86 – 707.68)	0.17 (0.05-0.53)

## Conclusion

PoCUS by clinicians reduced ED LOS and CT utilization in subsets of children with suspected appendicitis. This practice appears to be highly specific and safe when used to rule in appendicitis.

